# “Take me seriously”: A qualitative interview study exploring healthcare experiences of endometriosis patients

**DOI:** 10.1371/journal.pone.0323883

**Published:** 2025-05-16

**Authors:** Lara Brauer, Werner de Cruppé, Max Geraedts

**Affiliations:** Institute for Health Services Research and Clinical Epidemiology (IGVE), Philipps-University Marburg (UMR), Marburg, Hesse, Germany; UFSCar: Universidade Federal de Sao Carlos, BRAZIL

## Abstract

**Introduction:**

Endometriosis is a chronic disease associated with stigmatisation and delayed diagnoses. In order to create or improve positive healthcare encounters for patients, this study aimed to explore the experiences of women with endometriosis and to identify challenges and resources for these patients.

**Methods:**

Qualitative, semi-structured interviews were conducted to explore lived experiences of endometriosis patients. A purposive sampling strategy was used, and patients were included if they had a laparoscopically confirmed diagnosis and at least one contact with the healthcare system in Hesse, Germany. Interviews were conducted between May and August 2023, transcribed, and analysed according to an interpretive phenomenological approach using MAXQDA 24.

**Results:**

In total, 21 women aged 23–54 years (median: 32 years) were interviewed. Three themes were identified, with *ambivalenc*e emerging as the common essence of experiences. Patients reported that their experiences were influenced by the *role of the healthcare provider*, described as either a source of support or an inhibitor in the treatment process. Additionally, patients reported that *provider-patient-communication* was characterised by both trivialisation and dismissal of symptoms, as well as empathic, encouraging interactions. Limits in the healthcare system, such as the allocation of specialised care, and resources like increased awareness of endometriosis, represented *systemic influences on experiences*.

**Conclusion:**

This study provides an insight into the ambivalent nature of healthcare experiences from the perspective of endometriosis patients and contributes to a better understanding of patients’ needs in healthcare encounters, which may help to create a more positive healthcare experience for them.

## Introduction

Endometriosis is a hormone-dependent, chronic inflammatory disease characterised by the growth of endometrial-like tissue outside the uterus [1]. This can lead to pain (often menstrual cycle-related) and other symptoms such as bladder and bowel dysfunction, pain during intercourse, infertility or reduced fertility [1–3]. Also, a higher risk of certain cancers has been linked to endometriosis, e.g., a recently published 4.2-fold increased risk of ovarian cancer [4]. Exact prevalence rates are unknown, but it is estimated to affect between 2–10% of the female population overall [2]. The disease can become chronic even with optimal treatment, indicating that patients may need lifelong care [3]. The aetiology and pathogenesis of endometriosis is still poorly understood [1,3].

The disease can affect patients life considerably [5], particularly the quality of life, social contacts and work life [2], and is also associated with high levels of anxiety in patients [6]. It is known that patients often experience a lack of understanding from the healthcare system, stigmatisation of their pain and delayed diagnosis [5,7–9]. International research revealed clinical shortcomings and called for more practices to facilitate more encouraging contacts with the healthcare system [5,8]. However, to create or improve positive experiences for endometriosis patients, more research into their healthcare encounters is still needed [10].

In Germany, the precise healthcare situation of endometriosis patients or even reliable rates of endometriosis prevalence are not available due to a lack of data [11]. However, it is estimated that 10–15% of all women of reproductive age are affected and 40,000 women develop endometriosis each year [11]. On average, 2.3 years elapse between the onset of symptoms and the first contact with providers, a further average of 7.7 years elapse from the first contact before a diagnosis is made [12].

In general, the German healthcare system offers universal health insurance coverage for the population and a comprehensive benefits package with relatively low cost-sharing requirements [13]. It offers free choice of provider and is financed by statutory health insurance and private health insurance, which are mandatory for everyone registered or usually resident in Germany [13]. Furthermore, it is characterised by a relatively strict divide into outpatient care (with general practitioners and specialists), inpatient care (hospitals) and rehabilitative care [14].

Current German research based on national outpatient claims data has shown a continuously rising annual prevalence of diagnosed endometriosis in the period from 2012 to 2022, but clearly remaining below epidemiological prevalence estimates [15,16]. This suggests high level of underdiagnosis [15]. Although graduated evidence-based recommendations are given in the guideline *Diagnosis and Treatment of Endometriosis* [17], a malfunctioning or insufficient care seems to exist. This is particularly seen in delays in diagnosis, which are associated with provider-centred causes, but are also described as patient-related [18]. Currently, laparoscopy is the gold standard diagnostic test according to the named guideline [17]. Regarding surgical approaches, it is known that one third of surgical procedures are carried out in specialised, certified endometriosis centres and approaches used in those centres mainly follow recommendations of current guidelines in endometriosis care [19].

Notably, in Germany, little qualitative research has been conducted on experiences of endometriosis patients with healthcare encounters. So far, research is focused mainly on diagnostic delays, influences on the patients’ lives or on clinical aspects such as treatment options. Therefore, this study aimed to specifically describe the patients’ experiences of healthcare and its providers. The objective was to draw conclusions about the perceived current state of endometriosis healthcare from the patient’s perspective and to identify resources and challenges for patients to improve or create positive healthcare experiences.

## Methods

### Study design

This study used an exploratory qualitative approach, as this method is particularly suitable for investigating a phenomenon that has been little researched [20]. Interviews were conducted to explore the experiences with the healthcare system and its providers in the outpatient and inpatient sectors. Providers were defined as all inpatient and outpatient healthcare doctors and other medical personnel, health insurance companies or other relevant institutions for healthcare.

For the interviews, a phenomenological approach was used because it generates a deep understanding of a phenomenon, leads to in-depth interviews and identifies commonalities among participants [21,22]. This approach is particularly suitable when the focus is on analysing the essence of interviewees’ experiences of the same complex situation, as it aims to reveal both the structural and personal dimensions of the phenomenon by different individuals [20,23]. The overall aim is to abstract this universal essence from individual experiences, meaning the shared underlying structure, without including the expectations of the interviewer [21]. A research problem best examined with a phenomenological approach is one where it is essential to understand several individuals’ common experiences in order to devise practices or policies and/ or gain a deeper understanding about the characteristics of a phenomenon. Therefore, knowledge of these common experiences can be especially valuable for medical staff and policy makers [21].

This research method has already been used to study in-depth lived experiences related to complex phenomena in health services research in general [21] and specifically for interviews on experiences of endometriosis patients during healthcare encounters [10].

Given our interest in the underlying shared lived experiences of endometriosis patients during healthcare encounters, phenomenology offered the most appropriate methodological framework. It allowed us to identify the structures of experience that are common across participants, despite their heterogeneous medical histories. Our aim was to explore these in-depth shared experiences in order to understand the impact of healthcare encounters and to derive possible health policy improvements that would benefit patients or further support providers in dealing with endometriosis. We therefore preferred a phenomenological approach over grounded theory, which focused more on generating new theories from data and typically aims to develop a conceptual framework [21], which was not the main objective of our study. We also decided against a thematic analysis approach, which centres more on developing themes across cases but does not provide the same depth of insight into the structure of lived experience as phenomenology does [24].

A phenomenological interview typically contains open-ended questions in form of a semi-structured dialogue [20]. Thus, a semi-structured interview guide was developed from an analysis of the current research available on endometriosis care.

The *Consolidated Criteria for Reporting Qualitative Research* (COREQ) checklist was used to guide the reporting of study results [25]. This qualitative study is one part of a published study protocol [26] and has been approved by the Philipps University Marburg Ethics Committee (reference number: 23–14 BO).

As an example, the region of Central and Eastern Hesse, Germany, was examined. Central and Eastern Hesse, with a population of about 1.5 million (51% female) [27], was selected since it has not been studied in terms of the research questions and offered low-threshold access to patients and the opportunity to talk with patients face-to-face. This region contains clinics with gynaecology departments that treat endometriosis, including a specialised endometriosis centre.

### Sample

Purposive heterogeneous sampling was used to increase variation in patient characteristics [28]. We selected endometriosis patients using a maximum variation strategy in order to identify important shared patterns between patients emerging from heterogeneity [28]. To provide an information-rich case mix of different patient experiences, we varied patient age, date of diagnosis and area of residence (urban or rural) in our sample, as these are potential characteristics often associated with different patient experiences.

Inclusion criteria were a laparoscopically confirmed diagnosis of endometriosis (*ICD-10: N80 endometriosis* with all subcategories), at least 18 years old, German-speaking and resident in the included region for at least six months. Participants also had to have at least one encounter with the healthcare system in this region regarding their endometriosis.

### Recruitment

The recruitment period started on the 09 May 2023 and ended on the 04 September 2023. Patients were recruited through various methods: university mailing list, flyers in the gynaecology department of University Hospital Marburg, a regional self-help group, the institute’s website and verbal transmission. In addition, the *Endometriosis-Association Germany* published the call for participation on its website and on its social media (Facebook, Instagram, Twitter/ X). Interested patients contacted the first author via e-mail or telephone. During the first contact, it was ensured that patients met the inclusion criteria. With suitable patients, an interview was arranged at a time and location chosen by the patient. For face-to-face interviews, the premises of our institute or a location of their choice were offered. Also, virtual interviews were offered to minimise potential barriers to participation. Virtual interviewing, and therefore no travelling, increases flexibility for participants [29]. We decided against telephone interviews and in favour of virtual interviews, as they allow for contextual factors, such as eye contact and non-verbal interaction, as in face-to-face interviews [30].

### Data collection and setting

All interviews were conducted by the first author, who is female and attended three training sessions on developing, conducting and analysing qualitative studies prior to the interviews.

Before the interview, patients received detailed verbal and written information about the study and were informed that only experiences in relation to endometriosis will be part of the interview. Each patient provided verbal and written informed consent before the interview. Consent for publication of pseudonymised quotes for research purposes was obtained from each patient. Demographic data (e.g., age, diagnosis date, education level), interview location, length, and a pseudonymisation token were recorded.

As an icebreaker, an open-ended question was asked about how patients would generally describe their experiences with the healthcare system in relation to their endometriosis. Probing questions such as “Can you describe it in more detail?” or “Can you give an example?” were used to probe deeper. For example, questions in the interview guide were (complete interview guide: S1 Table):

- What has been your general experience with the healthcare system in terms of your endometriosis disease?
- Please describe your medical history in as much detail as you feel comfortable with.
- What positive and/ or negative experiences have you had in the course of your diagnosis and further care of your condition since your diagnosis?
- How do you think the German healthcare system should be changed in order to provide (even) better care? And what should be maintained?

One pilot interview was conducted to test the interview guide under realistic conditions. The patient provided written informed consent and gave feedback afterward. This feedback and the overall evaluation of the interview process were considered, and the pilot interview was included in the analysis due to its detailed and rich information.

The interviews were conducted by the first author in German between May and August 2023. Overall, there was great interest from patients in participating. New patients were continuously recruited until no new insights were reported in the last three interviews. After 21 interviews, data saturation was reached and no further participants were included. This is in line with the phenomenological approach, where a sample size of five to 25 participants is expected [21]. Interviews lasted an average of 36 minutes (*minimum*: 25 minutes, *maximum*: 65 minutes). Only the first author and the patient were present during the interview. In total, 12 face-to-face interviews were conducted, nine interviews were performed virtually. Two of the face-to-face interviews were conducted in local cafés chosen by the patients, 10 interviews were performed in a seminar room at our institute. There was no need to repeat interviews. Field notes were taken during and after the interviews. The interview audio was digitally recorded and transcribed verbatim. The first author initially transcribed and proofread the interviews alone. The transcripts were cross-validated by an experienced qualitative researcher from the first author’s extended network.

### Data analysis

The interviews were analysed using the interpretive phenomenological approach according to Creswell’s simplified version of Moustakas’ modification of the Stevick-Colaizzi-Keen method [21,22].

As phenomenology aims to exclude expectations of the interviewer, the first author reflected on her beliefs and experiences before data analysis to minimise potential bias during interpretation. The first author therefore reflected on her experience with the healthcare system as a woman and wrote down a short essay about her personal impressions and beliefs on provider encounters and diagnostic and treatment procedures. These experiences and assumptions were then tried to be set aside in order to be open to new perspectives during the analysis. This personal bracketing cannot be done completely, but can be seen as an effort to direct the focus on the participants [21].

First, interview transcripts were read multiple times to gain an overall sense. *Significant statements* were highlighted for each transcript and clustered into general, recurring *ideas*. These ideas were grouped into larger units called *themes*. For each theme, both a textural description (*what* happened) and a structural description (*how* it happened) were identified. Then, these descriptions were combined into a composite description of the phenomenon, representing the *essence* of the experience in phenomenology. This essence represents the culmination of a phenomenological approach [21].

Ideas were identified inductively from the transcripts and interpreted into general themes using MAXQDA 24. The first author performed data analysis separately. The results were then discussed and the interpretations evaluated with an experienced qualitative researcher from the authors extended network and with the co-authors.

## Results

### Patient characteristics

In total, 21 patients agreed to participate in this study, met the inclusion criteria and set a date for the interview. There were no drop-outs. The women were aged between 23–54 years with a mean age of 32.2 years (SD: 7.4) and a median of 32 years (Table 1). The date of diagnosis ranged from 1 month to 38 years ago at the time of the interview (*median*: 18 months). Six of the women had children. The classification of the patient’s highest educational qualification according to the *German National Qualification Framework* (DQR) ranged between 3 and 8 (*median*: 4), with *8* representing the highest qualification (doctorate), and *1* representing the lowest qualification (no degree) [31]. 13 women lived in urban areas, eight in rural areas. None of the participants were known to the interviewer.

**Table 1:** Patient characteristics.

Patient	Age	DQR	Area	Children	Diagnosed since	Interview place
**01**	28	4	Urban	0	1.25 yrs.	face-to-face
**02**	25	4	Urban	0	2.5 yrs.	face-to-face
**03**	28	4	Rural	0	6 mo.	face-to-face
**04**	54	8	Rural	2	38 yrs.	virtually
**05**	26	7	Urban	0	6 mo.	face-to-face
**06**	32	4	Rural	0	1.5 yrs.	face-to-face
**07**	34	7	Urban	0	1 mo.	face-to-face
**08**	38	4	Urban	1	3.5 yrs.	face-to-face
**09**	27	7	Urban	0	1,75 yrs.	face-to-face
**10**	41	7	Rural	2	3 mo.	virtually
**11**	23	4	Urban	0	1.5 mo.	face-to-face
**12**	36	3	Rural	0	2 yrs.	virtually
**13**	35	8	Urban	0	1 mo.	face-to-face
**14**	30	6	Urban	0	2.75 yrs.	virtually
**15**	37	6	Rural	2	8,75 yrs.	virtually
**16**	32	4	Urban	0	1.5 yrs.	face-to-face
**17**	32	4	Rural	1	5.75 yrs.	virtually
**18**	23	6	Urban	0	1.5 mo.	virtually
**19**	30	7	Urban	0	7 mo.	face-to-face
**20**	25	4	Urban	1	4 yrs.	virtually
**21**	41	4	Rural	0	1.5 yrs.	virtually

DQR, German National Qualification Framework; mo., months; yrs., years

Quotations in the results were translated meaningfully into English and denoted by patient number (e.g., Patient 01: P01). Filler words not necessary for understanding were removed from quotes. Further quotes, categorised by themes, can be found in S2 Table.

### Essence of the interviews

After analysis, three themes emerged as the main influences on the reported healthcare experiences: role of the healthcare provider, provider-patient-communication and systemic influences on experiences. These themes were combined into one essence: the great *ambivalence* experienced by endometriosis patients during healthcare encounters and with providers. This ambivalence describes the overall experience of the patients interviewed and was evident in each theme.

### Theme 1: Role of the healthcare provider

The role of healthcare providers was interpreted as central to the experiences of endometriosis patients. Providers were described as either facilitators or inhibitors in their care experience.

Providers who were seen as *facilitators* by patients were described as empathetic, reassuring, committed and helpful in providing access to care and referral to or consultation with specialists. The women expressed that these providers showed interest in the patient, were cooperative, accessible and they trusted their providers. Supportive providers were stated to suspect endometriosis fast. They took the time to talk to the woman in detail.

“*It happened very, very quickly and also the follow-up examination and (…) the follow-up treatment, I’d say, which I now have with him is just very, very good. So, I feel comfortable there because it happened so quickly*.” (P02)

Patients recounted being asked about painful periods or heavy bleeding and could ask questions about symptoms. These providers were described as having a broad expertise. According to the patients, this led to a quick diagnosis for them.

“*I was very satisfied. I told the gynaecologist about my pain and she said straight away that it could be endometriosis*. *She didn’t dismiss it at all, saying it was normal and suchlike, but addressed it directly.*” (P18)

After diagnosis, supportive providers were described as available, committed and providing close care. Patients felt “*in good hands*” and “*well cared for*” (P07).

Patients described their providers as *inhibitors* when there was perceived poor communication, when they reported disbelief, ignorance, or a lack of knowledge. Patient-proposed guideline-based treatments were reported as being rejected by the providers and they stated that access to further care was denied.

“*I was really persistent with the doctor I went to before (...). My best friend was even there with me, but he turned me down completely and really just wanted to give me the pill*.” (P01)

These experiences compounded the frustration and uncertainty felt by patients. The women described start-up difficulties with the providers, recurrent contacts until their diagnosis or provider changes. Patients talked about describing typical symptoms of endometriosis, but these were not addressed as endometriosis symptoms.

“*My gynaecologist never said anything about it. I always brought it up and said that I think it [the pain] goes beyond what my friends or other women tell me. And he just said, yes, you can take the pill or (…) your period is a painful process and that’s just the way it is. And yes, then I just put up with it, that’s just the way it is*.” (P03)

Other women felt that they had no space to explain their symptoms and felt dismissed. This would lead to misdiagnosis or late diagnosis.

“*I am a layman. (...) I have to be asked the right questions in order to get a proper diagnosis.*” (P15)

If the women were currently or in the past trying to have children, patients reported that the diagnosis was made because they could not get pregnant. In retrospect, they identified other symptoms of endometriosis, before trying to get pregnant, that had not previously been recognised by providers or themselves.

If their condition was not named as endometriosis, patients specified that providers recommended taking oral contraceptives (meaning the *pill*), or tried to treat the symptoms with plant-based medicine such as monk’s pepper (Vitex agnus-castus) or advised the patients to take painkillers. Opioids were also reported to be recommended for pain management.

In the absence of a diagnosis, access to further care or specialists was stated as difficult. The women felt rejected and had less trust in themselves and their provider. Even when they had a confirmed diagnosis, patients reported to feel like they are not looked after.

However, patient stated they made not only non-supportive experiences, but also met at least one supportive provider during their experiences with the healthcare system.

“*As long as you end up in the right place, with the right person, it’s completely fine*.” (P15)

It was also described that the role of the same provider could change over time from non-supportive to supportive. Patients described how their provider had researched their condition and treatment options and was now fully supportive.

### Theme 2: Provider-patient-communication

The provider-patient-communication was also described as influencing patients’ overall experience.

#### (Non-)Empathetic communication.

Patient-provider-communication was described on the one hand as lacking empathy. The women felt not seen or even disrespected by providers and wished for someone who at least partly takes away their fears and concerns. Communication characterised by sensitivity, patience and a better understanding of their individual situation was desired.

“*A bit more empathy overall would have been nice*.” (P14)

In contrast, other patients conveyed that they felt comfortable and seen. Providers were described as empathetic, open-minded and reassuring.

#### Information and education is power.

Participants illustrated that information and education about endometriosis is an important part for positive experiences. They praised the detailed education they received from their providers. Good information about their condition was cited by patients as the most important factor in positive care experiences.

“*Yes, I think that’s the most important thing, that you just get good advice*.” (P10)

Providers were recalled as informative and honest about what to expect from the diagnosis and medical findings. They felt taken by the hand and kept up to date, when providers took the time to talk to them in detail and referred them to specialists. Patients mentioned that providers educated themselves about endometriosis when identifying gaps in their knowledge.

“*That my gynaecologist is now undergoing further education. So that he is now (...) more aware of the topic. Not only with regard to me, but also to other patients that he may have previously overlooked*.” (P07)

Regarding laparoscopic surgery, the women recalled receiving detailed information about what would happen during and after the procedure. Also, the surgery was followed by a debriefing and the likelihood of future pregnancy was discussed and they verbalised hoping for follow-up rehabilitation to receive more education.

As another source of information, the so-called *Endo-App* was named, which can be obtained from the health insurance company, and was named as providing interdisciplinary information and advice on treatment options.

In contrast, patients also reported a lack of education. Not only did they have the impression of being insufficiently informed, but they also felt that the providers themselves had insufficient expertise. Patients indicated that they, and also providers, were unaware of patients’ rights and what they were entitled to, e.g., treatment options or follow-up treatment (“Anschlussheilbehandlung”). Providers and patients were initially unaware of specialised care such as endometriosis centres and of specific treatment options, such as an endometriosis pill. Patients wished therefore for more individualised advice, more communication and information. Additionally, they used social media to educate themselves, especially if they felt not fully informed by their provider.

“*We simply need more information*.” (P14)

Provider-patient contacts were also stated to be characterised by misinformation, e.g., incorrect information in their discharge letter, or they stated making poor decisions about their treatment because of a lack of information. For instance, they stopped taking contraceptives and symptoms worsened. Additionally, participants stated they were unaware of endometriosis, its meaning, or its symptoms because they had never heard of the disease before. They therefore desired more education from providers about painful menstruation and other symptoms, accompanied by a request for more awareness, more training for providers, and educational materials for patients.

“*But I had never heard of the term endometriosis before. I didn’t know it. My gynaecologist never mentioned it to me either. (...) Or that it even exists, that the uterus does something like that*.” (P10)

Additionally, patients described their providers as being left behind, when they apparently did not know what the diagnosis exactly meant, or detected cysts were not labelled as endometriosis.

Patients also declared assuming that their pain was normal and did not talk to their providers about it. Therefore, they did not blame their providers for not recognising their symptoms.

Furthermore, they noted a perceived lack of research and knowledge about endometriosis and understood that providers also lacked information. Consequently, they emphasised the importance of more support for providers.

In contrast, patients also said that endometriosis should only be mentioned when it has been diagnosed, and that no one needs to know about it before then.

#### Left alone.

Participants also talked about feeling left alone by their provider or the healthcare system. They criticised having to manage things on their own without any perceived support from providers. Particularly after surgery and diagnosis, they felt alone and unsupported and wished for more guidance.

“*You’re very much on your own in the whole healthcare system (...). You have to inform yourself; you have to decide for yourself what the right path is for you. And you just get few recommendations and little support*.” (P15)

Associated with this, patients called for more consideration of their personal circumstances and asked for self-help groups, so that they would feel less alone.

They also reported having to show a lot of personal initiative throughout the care process. They did not feel encouraged along the way or reported that they had diagnosed themselves and asked their provider directly if they might have endometriosis. They used search engines or social media for self-diagnosis, informed themselves before examinations, and researched treatment options. However, they also viewed self-diagnosis critically, acknowledging they are not medical experts. Also, patients stated they had to assert themselves to providers to get examinations, or urged providers to carry out diagnostic tests, wishing they did not have to run after treatments or tests. Patients (repeatedly) asked for referrals to specialised endometriosis centres or made appointments without feeling supported. This was seen as a general problem in the healthcare system.

Besides, the *Endometriosis-Association Germany* was declared as an additional helpful point of contact during and after the diagnostic process. There, the women felt seen and supported. Participants also said that it was alright to show personal initiative and that they were willing to help themselves. However, they wished for fewer perceived obstacles in their way.

“*That you get a bit of help, that you don’t have to sit down and help yourself - sure, you always have to help yourself. But simply getting help for self-help (...).*” (P11)

#### Patients’ competence with their own body.

Another factor was the extent to which patients felt they were seen as mature and competent by providers. Particularly the perception of (not) being taken seriously and whether the women’s pain was (not) seen as normal played an important role in their experiences. Every patient clarified, that being taken seriously by the provider was very important to them for positive healthcare experiences. They reported not feeling taken seriously especially when first seeing providers and before being diagnosed. After diagnosis, other concerns and symptoms were mentioned as not always being considered. They also stated that endometriosis was not being taken seriously enough in the healthcare system in general. Additionally, they indicated patients are not taken seriously by providers regardless of the condition, and perceived this as a typical problem for female patients. Not being taken seriously led to reported frustration, uncertainty about their future and a feeling of powerlessness. They wished for more acceptance and for their symptoms to be initially examined without scepticism.

“*Take me seriously or educate yourself, but don’t dismiss it*.” (P07)

In contrast, other patients recalled that they were directly taken seriously by their providers and that they were helped without hesitation.

“*With my gynaecologist, I had the feeling that she was taking it very seriously and that she was really trying to help me*.” (P13)

Another discussed issue was the trivialisation of pain. When the patients had the impression that their pain was dismissed, they felt not seen as mature patients or as competent in relation to their own bodies. Painful menstruations verbalised by the women were reported to be declared as normal. Patients stated being told that their pain was imagined and that they should not act up. They reported that providers labelled their pain as learned, or attributed the pain to stress, and solely referred them to painkillers. The women verbalised that they hoped for more awareness that menstrual pain is not normal, or only to a certain extent.

“*That they don’t tell patients that the pain is normal, because (...) it’s not normal. Maybe to a certain extent, but not to the point where you can no longer take part in everyday life*.” (P09)

In direct comparison with friends, participants have noticed that their pain was different, but also thought that their pain was normal. Hence, they did not talk about it with their provider or accepted it as normal.

Patients indicated that their experiences of being taken seriously or trivialising of their pain changed after their diagnosis. The time of diagnosis marked a turning point, after which they felt well cared for and further steps were initiated. They explained that having the diagnosis in black and white particularly helped them to be taken seriously.

### Theme 3: Systemic influences on experiences

The experiences were also influenced by structural factors of the healthcare system, interpreted as systemic resources or limitations.

#### Resources.

As an important systemic resource, particularly the *Endometriosis-Association Germany* and specialised endometriosis centres were named. Furthermore, patients reported that education and awareness of endometriosis had gradually increased in recent years. They perceived that the healthcare system is more aware of endometriosis and has become more competent. Patients also talked about health policy changes, which they supported.

“*I think it’s now on the radar*.” (P18)

Participants highlighted the fast process from symptoms to diagnosis and treatment, the easy access to hormonal treatment even without a diagnosis, or the possibility of taking a specific endometriosis pill.

Patients described surgery and minimally invasive laparoscopy as routine procedures in the hospitals, so they were confident in the expertise of providers. Consistency in treatment recommendations between different providers also created trust.

Patients highlighted the possibility of post-operative rehabilitation and the fact that a certain level of disability could be recognised by the authorities. The fact that suspected endometriosis can also be investigated by ultrasound or MRI, instead of undergoing surgery directly, was another resource mentioned.

Furthermore, the possibility of visiting fertility centres was emphasised if there was a desire to have children. A holistic approach to the disease (including, e.g., nutrition, osteopathy, psychotherapy) was also highlighted as a resource.

#### Limits.

Patients expressed as limits that the disease is still not present enough and health insurance companies could offer more to them. They wished for more counselling, more social support and more financial contribution to medication and holistic treatment options. They also talked about the perceived limited options for pain management and treatment of endometriosis. In this context, the women called for more research into the genesis and treatment of the disease. In particular, it was mentioned that a holistic approach should be further developed.

Difficulties in taking sick leave due to endometriosis were another limitation, which is why patients asked for opportunities to take some kind of menstrual leave. In this context, they spoke about the lack of recognition of endometriosis as a chronic disease and the limited right to only a minor recognised disability.

“*It is still not really noticeable for affected women that more is covered by health insurance. And something like menstrual leave is also not yet under discussion in Germany*.” (P05)

Also, patients pointed out that surgery is a major issue for them. Therefore, they hope for more non-surgical diagnostic options, e.g., saliva tests. Moreover, they suggested a proactive approach, such as questions at gynaecological check-ups, to raise awareness and speed up diagnosis.

Another named limit was the access to providers. Patients reported long waiting times for appointments and thus they wished for more capacity in the healthcare system. They expressed a need for more inpatient and outpatient providers. More local endometriosis specialists were suggested as a way to increase availability. The women hoped that this would lead to more detailed medical histories and perceived better care. They stated that finding a specialist was again a general problem of the healthcare system. Patients also mentioned the need for more regionally accessible specialised endometriosis centres, or a better allocation, and indicated that the distance to specialised care was too far. Also, they wished for more rehabilitation centres nationwide.

“*There is simply little expertise available locally. You always have to travel*.” (P15)

## Discussion

This study aimed to explore how women with endometriosis experience healthcare encounters in Germany using a qualitative phenomenological approach according to Creswell [21] with semi-structured interviews. The main finding is the *ambivalence* of endometriosis patients’ experiences with the healthcare system and providers. On the one hand, patients talked about meeting supportive, empathetic providers who were reassuring and took them seriously. This made them feel comfortable and satisfied with the received care. However, they also described encounters with providers who in their eyes did not offer appropriate care, dismissed them or trivialised their pain. The women felt abandoned and not seen as mature patients. Additionally, perceived limited access to specialised endometriosis centres and providers as well as resources such as increased awareness of endometriosis in the healthcare system were expressed.

To our knowledge, this is the first interview study on healthcare encounters from the perspective of endometriosis patients in Hesse, Germany. The results suggest a possible lack of knowledge or awareness of endometriosis and its treatment among providers. However, experiences appear to vary between providers depending on their attributes, resulting in patients feeling that they only received satisfactory care when they met the perceived “right” provider.

A Swedish study supports these findings, as it also revealed ambivalent healthcare encounters for endometriosis patients [10]. The encounters are described as double-edged, as all patients experienced both destructive and constructive contacts. Patients declared being treated with ignorance, but also being acknowledged [10], which is consistent with the findings of this study. The important role of the provider in the patient experience has already been shown for endometriosis patients in another study [32], but also in other pain-related conditions, e.g., degenerative cervical radiculopathy [33]. The providers were also described as facilitators or as inhibiting aspects for patients, with empathy, support and communication being particularly criticised [32,33].

The provider-patient-communication described in the interviews consisted of perceptions of not being taken seriously and of pain being trivialised. This trivialisation of pain, lack of understanding, not being taken seriously and the frustration is discussed in various qualitative studies on endometriosis or abdominal pain [5,7–9,34]. In particular women’s pain seems to be often dismissed as “normal” dysmenorrhea [35], which can lead to feelings of helplessness, guilt and can influence future healthcare encounters [34]. This normalisation of women’s pain can be seen in the context of a researched phenomenon called the *Gender Pain Gap*, which refers to undertreated and less understood pain in women [34]. However, legitimacy was shown to be crucial for patients with chronic pain. Therefore, women described healthcare encounters as a struggle for both patients and clinicians [36]. Conditions where pain is a main symptom are dominated by women, however, they are often questioned as patients. Those conditions are often underexplored and pictured as challenge for medicine. Besides, women with chronic pain receive less and less effective pain medication and more referrals to mental health providers [36]. Evidence of gender bias in healthcare encounters or prescribed medication is documented for chronic pain [36] and also for other conditions, e.g., stroke care [37]. The root of this gender bias in healthcare practice may lie in the organisation or in biased elements of established medical knowledge [38]. In addition, the subjectivity of pain assessment by healthcare providers makes the treatment or experience of pain prone to gender norms [36]. This bias applied to endometriosis care could explain the neglect and normalisation of pain by providers reported in our interviews, as well as delayed or incorrect diagnoses and treatment decisions favouring oral contraception or herbal medicine.

The reported neglect of patient’s symptoms by healthcare providers is referred to as *medical gaslighting* [39]. Experiences of medical gaslighting particularly affect the availability of care and care-seeking behaviour of women [39]. If they have a trusted provider and feel safe, they are more likely to seek care. In contrast, if they experience medical gaslighting, they would not seek care in the future, feel ashamed and dismiss their symptoms themselves. This contributes to a decreased health literacy in relation to “normal” menstruation [39]. However, the term medical gaslighting is contentious and not only an endometriosis issue, but is also discussed in other conditions, e.g., long-covid [40]. The problems in provider-patient-communication reported in the interviews may be explained partly by the Gender Pain Gap and medical gaslighting.

Not only did provider-patient-communication in general seem to influence the encounters, but also the individual experience and knowledge of the providers. Providers who were described as having experience or expertise in endometriosis were perceived as being helpful and reassuring, and patients felt better cared for and were more satisfied. With providers described as less experienced, patients reported having to be more persistent, experiencing more disbelief and having to ask several times for diagnostics or treatment. These patients often self-diagnosed before receiving their official diagnosis. For self-diagnosis, in particular social media or patient-led self-help groups were a source of information and contributed to self-diagnosis. After diagnosis, the *Endo-App* was mentioned in the interviews as an important source of information. The main source of information about the condition, its diagnosis and treatment for the patients was the *Endometriosis-Association Germany*, a patient-led self-help organisation. Patients stated that the association helped them with all aspects of their medical history, especially with navigating the healthcare system, feeling less alone with their condition, and with joining local support groups. Patients can also connect with each other online through their social media, especially Instagram. These online and offline interactions with other patients were highlighted by the patients as an important aspect.

A meta-synthesis including studies from 2004–2018 found that the reported experiences, such as providers insufficient knowledge and treatment of women with ignorance and trivialisation of symptoms, seem to persist over time [8]. Another systematic review supports these results, stating that poor experiences for women, including medical gaslighting, have been identified in qualitative research over the past decades (1999–2018), suggesting that there has been little improvement [41]. One explanation for the perceived lack of empathy for endometriosis patients may be that interviewed providers stated in a study that they felt inadequately trained to address the psychosocial aspects of endometriosis, and half of the gynaecologists did not consider it their responsibility [42].

However, in this study, also a perceived improvement in endometriosis care was reported. This improvement was also seen in current research, which interpreted increased awareness of endometriosis due to higher prevalence rates [15,16].

As the women reported more acknowledgement of their pain after diagnosis, they talked about their diagnosis as a turning point in healthcare encounters. The division of encounters into before and after diagnosis of endometriosis patients was also revealed in previous interviews [7]. However, after diagnosis, the patients also mentioned the trivialisation of their pain and stigmatisation, which may indicate that there seems to exist a lack of understanding or knowledge of the disease by providers. Other aspects of care, such as a lack of treatment options for patients or the fact that a different provider diagnosed the patient (e.g., in the certified endometriosis centres), may also be reasons why a diagnosis did not always lead to perceived better care.

A current toolbox to collect high-quality patient experience data supports the thesis that diagnosis can be a turning point, as the authors divide the experiences into three parts: life before a diagnosis, experiences getting a diagnosis and experiences living with a diagnosis [43]. These domains were all reported by patients in this study, indicating that the interviews covered all important aspects of patient experiences.

Furthermore, the stated recurrent contacts are in line with estimated recurrence rates of 50–80% due to necessary healthcare encounters, even after surgical treatment [44]. However, interviewed patients reported recurrent encounters and provider changes to receive their diagnosis or due to the fact that they were searching for a perceived helpful provider. It is already known that long pathways to an endometriosis diagnosis result in high costs and increased use of the healthcare system [18]. These increased healthcare encounters appear to be partly avoidable and illustrate the economic impact of endometriosis on the healthcare system.

As shown, experiences with providers and provider-patient-communication are described very similarly internationally, despite differing healthcare systems. This suggests that factors like stigmatisation or expertise may be more decisive for endometriosis care than the healthcare system structure.

Our interviews also revealed a need for more holistic endometriosis care. An existing holistic model, the Chronic Care Model by Wagner, has been tested for endometriosis care and complemented by multidisciplinary providers [45]. This model would allow for a patient-centred approach with different providers, and identified the patient-gynaecologist interaction as central to coordinating care, which is consistent with our findings. A recent review also concluded that a multidisciplinary biopsychosocial care model is needed to manage heterogeneous symptoms, possibly in the form of multidisciplinary endometriosis centres [46]. It was suggested that such multidisciplinary centres could consist of gynaecologists, pain specialists, physiotherapists, psychologists, nutritionists, but also general practitioners to provide holistic care [46]. These providers were also requested by patients in our interviews. In agreement with further previous research [5,42], this study supports the importance of enhancing clinical guidelines and promoting holistic treatment practices to optimise endometriosis healthcare.

The interviews also highlight the importance of investigating endometriosis, with patients calling for more research into aetiology, treatment, and non-surgical diagnostic options. Research into endometriosis itself and into healthcare encounters needs to be more emphasised and funded by policy. Regarding diagnosis, laparoscopy is currently gold standard in Germany [13], but this is being challenged by European guidelines [2], supporting the request for alternative diagnostic options, such as, e.g., diagnosis via a salvia test. Several policy interventions and recommendations for healthcare providers could be developed based on our results to ensure quality and to address inadequate or insufficient endometriosis care. For example, funding and resource allocation for procedures and treatments should be politically prioritised. Also, more education and support could be provided to healthcare professionals, particularly to improve the described inadequate provider education and general knowledge about the disease, to potentially speed up diagnosis and to enhance communication with patients. For instance, specific training for providers in communicating with endometriosis patients could be developed, covering not only educational but also psychosocial aspects. Existing chronic care models and multidisciplinary approaches, as described above, could be part of such training. Education on gender bias and gender-sensitive care, as well as the management of chronic pain in endometriosis patients, could also be helpful for providers. Developing such training might increase providers’ knowledge, raise awareness of aspects of endometriosis care that are important to patients, and ensure a more satisfying experience for both patients and providers.

This study adds new insights into endometriosis patients’ experiences and identifies three key themes influencing encounters. Further research is needed with both patients and providers. In order to understand providers’ perspectives on endometriosis healthcare, their experiences could be further analysed. Additionally, research on provider-patient communication and the role of providers in the care process could help enable more positive experiences for patients. Further education and support for both patient and provider could be made available, e.g., through enhancing clinical guidelines. Also, various interventions could be developed to ensure positive experiences, e.g., specific training in communication with endometriosis patients.

One limitation of this study is that the interviews were conducted regionally in Hesse, Germany as an example area. This region was chosen due to low-threshold access to patients and the opportunity to talk with patients face-to-face. It might be possible that regional factors also influence the experiences of endometriosis patients and therefore, the results of this study. Although the general structure of the German healthcare system is comparable across all counties, regional differences in healthcare delivery or access to services may lead to variation in experiences. While we assume that similar experiences are likely in other German regions, or even in other countries with a different healthcare system structure, any generalisation of the findings needs to be done carefully.

Regarding the demographic variety of patients, it is notable that few patients with a lower educational qualification participated in our interviews. This may result in a potential bias in representation. For example, socioeconomic status could influence the healthcare experience and affect patients’ choice of hospital. Patients with higher status may have fewer difficulties in choosing or travelling to care, whereas those with lower status may lack access or information [47]. This needs to be considered for interpretation and generalisation of the results, as the experiences of patients from lower socioeconomic backgrounds may not be fully represented. Additionally, it is notable that only three participants were older than 40 years in our sample and the majority was between 23–38 years old. As age-related differences can influence patients’ experiences and interactions with healthcare providers [48], and peri-, post- or menopausal women with endometriosis in particular may have different healthcare needs, their underrepresentation in our sample may affect the generalisability of the findings due to this demographic factor.

However, participants varied in terms of date of diagnosis and general age range (as defined in the purposive sampling strategy), as well as in the reported experiences, so we assume that we still obtained diverse results across these characteristics.

Another limitation is a potential bias in studies based on patient reporting, as patients might want to talk more about poor experiences rather than what they perceived as good care. It is possible, that positive experiences are underrepresented in these findings. However, we were aware of this before and during the interviews and formulated neutral, open-ended main questions and probing questions to ensure that patients had space to talk about positive experiences during the interviews. In addition, the described ambivalence in the experiences and the explicitly named positive experiences show that a broad spectrum was reported. Furthermore, according to the Thomas theorem, qualitative findings may be subjective, but they are real to the participants, and therefore have real consequences for them [49]. This implies that what patients recalled in our interviews may be biased, but can still be considered valid.

A strength of this study is the used phenomenological approach, which generated detailed interviews and enabled the identification of dynamics affecting patient experience with healthcare. The sample size of 21 women was sufficient to achieve in-depth analysis, as it is at the upper end of the recommended range of five to 25 participants, with saturation typically being reached at this sample size in phenomenological research [21]. In the final three interviews, no fundamentally new insights were generated, indicating that saturation was reached after 21 patients. Given the diversity of age, medical history and healthcare experiences of the patients, we were able to capture a wide range of data and themes relevant to patients with endometriosis. Additionally, the COREQ checklist [25] was used to ensure the quality of reporting of this study.

## Conclusion

This study contributes to the limited research on endometriosis patients’ experiences of healthcare encounters. Ambivalence between providers and their communication, or with the same provider over time, affects patient experience. Trivialisation of pain, not being taken seriously by providers and lack of education were described as the main causes of perceived poor experiences, whereas supportive, empathetic providers were portrayed as a positive impact on patients’ experience. In addition, systemic factors such as limited access and allocation of specialised care, but also a perceived increased awareness of endometriosis in the healthcare system, influenced the experiences. The results could have an impact on healthcare encounters of endometriosis patients, as their needs may be better understood. Further research is needed with patients and providers to support them before, during and after diagnosis.

## Supporting information

S1 Table Interview guide. (PDF)

S2 Table Themes and quotes. (PDF)
