# Supplementary material for: “Take me seriously”: A qualitative interview study exploring healthcare experiences of endometriosis patients
Source: PLoS One. 2025 May 16;20(5):e0323883. doi: 10.1371/journal.pone.0323883 (PMC12083814; doi:10.1371/journal.pone.0323883)
Supplement: S1 Table — (PDF) [file pone.0323883.s001.pdf]

Interview guide: semi-structured interview on healthcare system encounters and diagnosis of endometriosis

**Age**	
**Highest qualification**	
**County**	
**Date of diagnosis**	
**Children**	
**Interview token**	
**Date**	
**Place**	
**Duration in mins**	
**Declaration of consent**	

I. Opening question

Dear Mrs. XY, I would like to interview you today about your experiences with the diagnosing process and your contacts with the healthcare system, i.e. with doctors, hospitals, other healthcare facilities or healthcare providers in relation to your condition. What has been your general experience with the healthcare system in terms of your endometriosis disease?

II. Main part

1. Please describe your medical history in as much detail as you feel comfortable with.
2. Which medical specialists and healthcare facilities have you visited in relation to your condition? (e.g. gynaecology, general medicine, endometriosis centre, etc.)
3. What positive and / or negative experiences have you had in the course of your diagnosis and further care of your condition since your diagnosis?
4. How would you rate the expertise of the physicians and non-physicians with whom you have had contact during the course of your disease?
5. How sensitised do you think the healthcare system is to the disease in general?
6. Would you do anything differently today with your current knowledge and diagnosis than you did back then?

7. Which services and facilities did you find particularly helpful?
8. How do you think the German healthcare system should be changed in order to provide (even) better care? And what should be maintained?
9. How would you like your contacts with the healthcare system in relation to your condition and yourself to be in the future?

Key questions

1. Please describe your medical history in as much detail as you feel comfortable with.
2. What positive and / or negative experiences have you had in the course of your diagnosis and further care of your condition since your diagnosis?
3. How do you think the German healthcare system should be changed in order to provide (even) better care? And what should be maintained?

Narrative impulse and probing questions

1. Would you like to explain the last situation you described to me in more detail?
2. Can you give me an example of how you experienced XY / what does XY means to you?
3. What happened after the situation you described to me?
4. How did you feel? / What did you feel / think in this situation?
5. Can you give an example?
6. Can you describe it in more detail?
7. What happened next?
8. Can you remember a specific situation? Would you describe it to me?

(Critical) enquiry and redirection to the topic of the interview

1. You just said XY. I probably understood that differently. Would you like to explain to me again how you view this point?
2. Did you mean that ...?
3. Do I understand you correctly if...?
4. This aspect is very interesting; however, I think it takes us too far away from my actual question. May I ask you again what exactly you mean by XY?
5. Thank you for this detailed description. What would you say is the most important aspect of question XY for you?

III. Summary and review

1. We are slowly approaching the end of our interview. As a final question, I would like to ask you: What would you like to tell people who are not affected by endometriosis about your condition and your journey and life with it?
2. We have now reached the end of the interview. Is there anything else you would like to add?
3. The main message I take away from the interview with you is that …
4. Thank you very much for your time and openness in sharing your story with me.

IV. Outlook

In the course of my doctoral thesis, I will write down and analyse what was said in the interview. This means that I will describe the similarities that all participants have when experiencing the same phenomenon in order to be able to make a general statement about the experience. The collected data will be analysed in such a way that the content is reduced to significant statements and quotes and then categorised into themes. These important statements are then published in a research paper. However, no conclusions can be drawn about you as a person. Do you have any questions about the evaluation process or the privacy of your data? If you are interested in the published results, I will be happy to send them to you.
