# Supplementary material for: “Take me seriously”: A qualitative interview study exploring healthcare experiences of endometriosis patients
Source: PLoS One. 2025 May 16;20(5):e0323883. doi: 10.1371/journal.pone.0323883 (PMC12083814; doi:10.1371/journal.pone.0323883)
Supplement: S2 Table — (PDF) [file pone.0323883.s002.pdf]

MAIN THEMES	SUB THEMES	QUOTES
**ROLE OF THE HEALTHCARE PROVIDER**	Role as Facilitator	• "*She [gynaecologist] is just great and answered all my questions and also explained all the treatment options.*" (P11)<br>• "*I can get in touch at any time if I have any symptoms.*" (P06)<br>• "*I've been coming back every six months now. He keeps a very close eye on me.*" (P11)<br>• "*We are now sailing in the same waters. And that's why I feel quite in good hands there now. Yes, but there also were start-up difficulties.*" (P07)
	Role as Inhibitor	• "*The first one didn't take my complaints seriously at all. The second one took them seriously, but also didn't think about endometriosis.*" (P05)<br>• "*I have to say, my gynaecologist (…) never asked me any questions, 'How much blood are you losing, how long does the pain last?' It's always like 'It's normal, pill'.*" (P05)<br>• "*Then I took... Tilidine. Novalgin. Buscopan. (…) So that I came out at 12, 13, 14 pills a day. (…) I realised that I (...) had this pain for a long time. But no doctor had ever really paid attention to it. In the meantime, I've been to 4 or 5 gynaecologists and everyone said it's normal, take painkillers.*" (P20)<br>• "*The gynaecologist doesn't do anything about it. She also doesn't really ask me when I come in for my normal check-up: 'Are you having problems again, or is something wrong?'.*" (P15)"

**PROVIDER-PATIENT-COMMUNICATION**	(Non-)Empathetic Communication	• "*That I don't have to feel like I'm just some part of an assembly line."* (P21)<br>• *"And then he said to me: 'Now would you stop crying? That's not a very grateful reaction to the surgery.'."* (P01)<br><br>• *"But I always had the feeling, (...), that they were all very approachable and empathetic."* (P10)<br>• *"I also felt very comfortable there (…). But even after the operation she said here is this and this and here you can see this (...), very empathetic in a human way."* (P11)
	Information & Education is Power	• "*My gynaecologist was able to show me a model and then explained directly where the cells accumulate and what they look like and what they do. (…) He was able to give me the information he had (…) very well*." (P02)<br>• "*The surgery steps (...), what exactly they're looking at, what they expect (…). They also said very clearly what it would be like with pregnancies. Simply that you were taken by the hand a bit and knew okay, this is my diagnosis now and this is what the path looks like*." (P06)<br>• "*I felt I was getting good advice, let me put it that way. From my gynaecologist, as well*." (P18)<br><br>• "*But I never received any information about whether I had any rights or anything else.*" (P17)<br>• "*There was simply no open communication or a lack of information from the doctors. (...) Somehow, it's also a bit scary that you get so little information about it, even though I now know that it's a very common illness."* (P19)

	• "*Because I was always of the opinion that it comes from the uterus. When it is no longer there. Nothing more can happen. But that's also something that was simply based on poor knowledge and poor information*." (P15)
Left alone	• "*You only get the diagnosis and have to deal with it yourself. (…) In other words, I was basically left completely alone by the gynaecologists*." (P17)<br>• "*That's actually the most negative thing (…), that I was left very alone at such a young age*." (P12)<br>• "*And then it was the case (...), that I simply checked out myself where there might be experts nearby. And then I saw: oh great, there's an endometriosis centre (...). I actually made an appointment there on my own and only asked my gynaecologist afterwards 'Can you write me a referral?'. So, it was this way and not the other way around*." (P13)
Patients Competence with their own Body	• "*In any case, that you are simply taken seriously. Not being portrayed as a hypochondriac (...). I think that's a very, very, very important point, that you feel taken seriously*." (P06)<br>• "*That you just give up at some point due to frustration because you're not getting anywhere and because you're not being taken seriously*." (P03)<br>• "*You get used to it at some point and think the pain isn't real because it's always dismissed. (…) It's always dismissed with 'All women have it and if you can't cope with it, you just have to take the pill' and it's been a recurring theme throughout the last ten years of visiting gynaecologists*." (P05)<br>• "*I also had many moments when I really doubted myself. I was also ashamed of it and was often told: 'Don't act up like that, every woman has it'*." (P12)

		• "*With my gynaecologist, I had the feeling that she was taking it very seriously and that she was really trying to help me. (...) People who first believe you that something is wrong or not quite right and then help you as much as they can, are particularly helpful*." (P13)<br>• "*Especially when you have the diagnosis in writing, you will be taken seriously. But only when the diagnosis has actually been made.*" (P16)<br>• "*I think the positive experience was when you had the diagnosis and you were taken seriously. (...) Because then it was there in black and white*." (P09)
**SYSTEMIC INFLUENCES ON EXPERIENCES**	Resources	• "*I believe that healthcare policy also has an eye on this at the moment. So, a lot is happening*." (P19)<br>• "*It's now a routine procedure for the doctors themselves, so there's not much that can go wrong, especially because it's so minimally invasive, which I think is good*." (P11)
	Limits	• "*This obstacle of having a surgery is really high. (...) I did it back then because I really had this pain and (…) something had to be done and I just wanted to be sure (...). But it was still a very huge obstacle for me*." (P09)<br>• "*I would say that, as a patient, I know more about it than the people around me. That includes all the doctors and therapists (...). I believe that the knowledge that the disease exists, is everywhere. But the details. And even recent research results (...) are very rarely known*." (P21)<br>• "*That you have to wait for ages. I thought that was really bad. Of course, you're not considered an urgent patient, I can understand that, but (...) a year is quite a long time*." (P19)
